# Trisecting representational states in short-term memory

**DOI:** 10.3389/fnhum.2013.00796

**Published:** 2013-11-26

**Authors:** Derek Evan Nee, John Jonides

**Affiliations:** ^1^Helen Wills Neuroscience Institute, University of CaliforniaBerkeley, CA, USA; ^2^Department of Psychology, University of Michigan, Ann ArborMI, USA

**Keywords:** working memory, prefrontal cortex, medial temporal lobe, attention, long-term memory

## Abstract

The ability to hold information briefly in mind in the absence of external stimulation forms the core of much of higher-order cognition. This ability is referred to as short-term memory (STM). However, single-term labels such as this belie the complexity of the underlying construct. Here, we review evidence that STM is an amalgamation of three qualitatively distinct states. We argue that these distinct states emerge from the combination of frontal selection mechanisms (often considered the domain of attention and cognitive control), medial temporal binding mechanisms (often considered the domain of long-term memory, LTM), and synaptic plasticity. These various contributions lead to a single representation amenable to elaborated processing (focus of attention), a limited set of active representations among which attention can be flexibly switched (direct-access region), and passive representations whose residual traces facilitate re-activation (activated LTM). We suggest that selection and binding mechanisms are typically engaged simultaneously, providing multiple forms and routes of short-term maintenance. We propose that such a framework can resolve discrepancies among recent studies that have attempted to understand the relationship between attention and STM on the one hand, and between LTM and STM on the other. We anticipate that recent advances in neuroimaging and neurophysiology will elucidate the mechanisms underlying shifts and transformations among these representational states, providing a window into the dynamic processes of higher-order cognition.

## INTRODUCTION

At the center of nearly all deliberative processes is short-term memory (STM)^1^. STM involves the retention of information in the service of ongoing cognition, usually lasting on the order of seconds. STM is used to hold in mind options when making choices such as what to eat at a restaurant or whom to pick in a fantasy football draft. It is used to keep track of traffic when navigating a car or crossing the street. It is used to comprehend this very text and store goals for upcoming tasks. As a result of its far-reaching impact, an appropriate model of STM is essential for understanding cognition.

The importance of STM is underscored by the relationship between variation in the capacity of STM and variation in higher-order cognitive abilities. For example, STM capacity predicts substantial variance in reasoning, problem solving, reading, language comprehension, and fluid intelligence (Daneman and Carpenter, 1980; Carpenter et al., 1990; Just and Carpenter, 1992; Daneman and Merikle, 1996; Fukuda et al., 2010). Moreover, intelligence has been shown to rise as STM capacity is increased through training (Jaeggi et al., 2008). STM capacity is compromised in psychiatric disorders such as schizophrenia (Gold et al., 2003), and these reductions are predictive of a wide-array of cognitive impairments (Johnson et al., 2013a). Hence, the amount of information that can be held in STM is a critical determinant of cognitive function.

While the importance of STM is without dispute, how to determine its capacity has been controversial. It is well-known that initial estimates suggested that 7 ± 2 items could be held in STM (Miller, 1956), which was based upon tasks that required simple repetition of digit strings. However, more complex tasks that require concurrently holding items in mind while processing other information have subsequently grown more popular (Daneman and Carpenter, 1980; Turner and Engle, 1989) as have tasks that require the detection of changes in arrays of visual objects (Luck and Vogel, 1997, 2013). Critical to these tasks is the minimization of strategic processes that might chunk multiple items into a single representation thereby rendering the number of maintained items ambiguous. When chunking is effectively minimized by concurrent processing or brief retention intervals, capacity is typically estimated to be 4 ± 1 items (Cowan, 2001).

Although a capacity limit of 4 ± 1 items is commonly observed across a broad range of tasks, evidence suggests that not all items held in mind are of equal status. In many tasks, a single item among the 4 ± 1 appears to hold a privileged position, one that makes it more accessible than other items. One task that reveals this involves rapid serial presentation of items followed almost immediately by a recognition probe (McElree and Dosher, 1989; McElree, 2006). In this task, retrieval and decision processes are carefully controlled by a response deadline. Varying the duration of the response deadline on a trial-by-trial basis enables the ability to track the rate at which information about the correct response accrues. Using such a procedure, it has repeatedly been shown that the most recently presented item can be accessed unusuallquickly while the rate of retrieval of all other items remains relatively constant (McElree and Dosher, 1989; McElree, 2006). Interestingly, if subjects are trained to pace rehearsal after all items have been presented, the most recently *rehearsed* item is accessed unusually quickly rather than the most recently *presented* item (McElree, 2006). These data indicate that speed of access is due to an item’s status in STM, ratherthan to its recent physical presentation. In other tasks, it has been shown that repeated processing of the same item held in STM is greatly facilitated, while switching processing to another item held in STM incurs a substantial cost in time (Garavan, 1998; Oberauer, 2002). These data suggest that although multiple items may be held in mind concurrently, at a given moment, a single item maintains a uniquely accessible status. Such considerations have led to proposals that multiple, qualitatively distinct representational states exist in STM (Oberauer, 2002; Jonides et al., 2008; Oberauer, 2009). We elaborate one such model next. We begin by reviewing prior behavioral evidence for the model and then detail neural data that will lead to a new conceptualization of the underlying neural systems.

## THREE-STATE MODEL OF MEMORY

**FIGURE 1:**
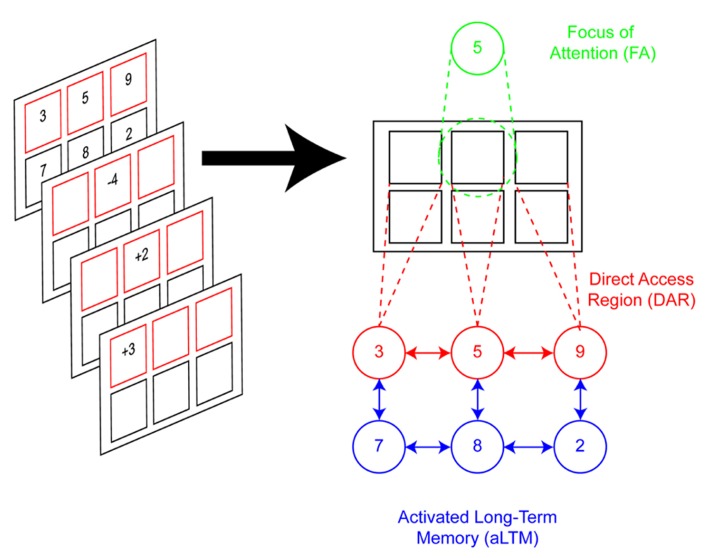
**Three-state model of memory.** Left: illustration of the task used by Oberauer (2002) to test the three-state model of memory. Participants hold in mind two sets of digits. Red frames indicate the active set whose digits are candidates for processing (e.g., top set). Black frames indicate the passive set, which is recalled at the end of the trial, but not the subject of operations. Mathematical operations are applied to the active set thereby updating its contents. In this example, “-4” is applied to “5,” resulting in “1.” Subsequently, “+2” is applied to the result, yielding “3” and so on. At the end of the trial, all digits are recalled. In this example, going left to right starting from the top, recall would be “6,” “3,” “9,” “7,” “8,” “2.” Right: depiction of the representational states of STM according to the model. The modeled scenario reflects the moment at which “-4” is presented. The cue “-4” draws the focus of attention (FA) to its corresponding frame. The number associated with that frame, “5,” is recalled through location-digit bindings. All location-digit bindings for the active set are maintained through the direct-access region (DAR). It is assumed that items are also inter-associated with each other, as well as other items that are not actively maintained (e.g., passive set). The passive set, which is not contextually bound, is held in activated long-term memory (aLTM).

Based on the evidence reviewed above, Oberauer (2002) proposed a three-state model of STM (**Figure 1**). First, the model distinguishes information that is actively held in mind from information that is passively maintained. Passively maintained information includes residual traces of representations that linger either due to recent presentation or to associations with actively maintained information^2^. Oberauer referred to this passive state as activated long-term memory (aLTM). aLTM is presumed to be the source of phenomena such as priming and proactive interference. By contrast, active maintenance involves binding information to a context. Contexts may include temporal details (e.g., the current trial in an experimental task) and/or other cues that may be associated with the maintained items (e.g., originally presented color or location). The active maintenance of these contextual bindings makes the information directly accessible through an appropriate contextual cue. The combination of the items and their bindings is referred to as the direct-access region (DAR). Finally, among the DAR bindings, a single item is selected for additional processing. It is this selected item that is amenable to computational and transformational processes (e.g., mental arithmetic). This privileged item is referred to as the focus of attention. Hence, information in STM may be considered to be in one of three states: aLTM, DAR, or focus of attention.

To test the model, Oberauer (2002) studied the task depicted in **Figure 1**. On each trial, subjects were presented with two sets of digits each associated with a different frame. The size of each set was varied independently between one and three items. Either one or both sets of frames were highlighted in red to denote that they were candidates for processing. For simplicity, we will consider only cases in which one set was highlighted. In this case, the highlighted set was deemed the active set while the non-highlighted set was deemed the passive set. Afterward, a series of simple mathematical operations appeared in the frames of the active set, one at a time, and subjects were required to apply the operation to the corresponding number and update their memory with the result. At the end of the trial, subjects recalled all of the updated digits. Two aspects of the task were critical: (1) subsequent operations could be applied to either the same frame or a different frame. In the latter case, a switch cost was hypothesized that would reflect the cost of shifting the focus of attention. (2) The sizes of the active and passive sets were independently varied. Only the active set was hypothesized to be bound in the DAR while the passive set was hypothesized to remain in aLTM. Consistent with research indicating that searching information in STM slows as more items are added to STM (Sternberg, 1966), Oberauer (2002) predicted an active set size effect such that updates would be slowed with higher active set sizes. Effectively, each update required a search of the DAR – the more items bound in the DAR, the longer the search. By contrast, no effect of the passive set size was anticipated since passive items should be maintained in aLTM, but not the DAR. Furthermore, the cost of switching the focus of attention was also predicted to increase with the size of the active, but not the passive set. This is because the focus of attention should only shift among items in the DAR. The data confirmed all of these predictions providing evidence for the three-state model (see also Oberauer, 2005).

Alternative models can largely be considered a subset of the three-state model. Cowan (1995) suggested a framework which does not distinguish the DAR and focus of attention. Instead, the focus of attention is thought to consist of 4 ± 1 items, all of which have an equivalent status. However, it is unclear how this model accounts for the costs of switching between different items held in STM. By contrast, McElree (2006) hypothesized a single item focus of attention, but no intermediate state between the focus of attention and aLTM. In this case, it is unclear how to account for the active, but not passive set size effects. Hence, two-state models appear unable to account fully for the various results described by Oberauer (2002, 2005).

As the example above illustrates, it is often the case that information is held in STM in the service of rule-based processes that include condition and response bindings (e.g., arithmetic). While the three-state model was originally formulated to accommodate declarative content, it has recently been updated to include procedural content, as well (Oberauer, 2009). Under this framework, procedural memory has an analogous three-state system with costs in switching responses analogous to costs in switching the focus of attention, and costs in switching sets of condition-action bindings analogous to costs in switching sets of item-context bindings in the DAR. It is then hypothesized that declarative and procedural STM interact such that the focus of attention provides input for condition rules while the output of those rules can be subsequently input into the DAR (Oberauer et al., 2013). Hence, the three-state model provides an account of dynamic aspects of STM, laying the groundwork for understanding complex cognition.

## NEURAL EVIDENCE FOR A THREE-STATE MODEL OF MEMORY

In a previous review, we hypothesized neural mechanisms that could produce a three-state signature in behavior (Jonides et al., 2008). At the time, we theorized that frontal areas provide top-down bias on posterior cortices whose activity reflects the neural representation of information in the focus of attention. Concurrently, the medial temporal lobe (MTL) binds information to its context enabling a basis for context-based retrieval, as well as laying the groundwork for new long-term memories (Schon et al., 2004; Ranganath et al., 2005). We conjectured that only information in the focus of attention is instantiated by active neural firing. Information outside of the focus of attention was thought to be sustained by rapid short-term synaptic plasticity (Zucker and Regehr, 2002; Mongillo et al., 2008). Furthermore, we hypothesized that the focus of attention can be flexibly deployed among all representations that share a given context in order to refresh those representations continually. In this way, all items linked to a context via the MTL reflect the DAR, and the focus of attention is cycled amongst them to maintain their fidelity. Items not linked to the currently relevant context were hypothesized to fade gradually as a result of interference processes (but see Zhang and Luck, 2009). The residual synaptic traces of such items were theorized to correspond to aLTM. This produced a layered framework wherein each successive state added a layer of neural instantiation (aLTM: synaptic plasticity, DAR: synaptic plasticity + contextual binding, focus of attention: synaptic plasticity + contextual binding + active cortical firing). In work that followed, we and others tested the model using functional magnetic resonance imaging (fMRI).

**FIGURE 2:**
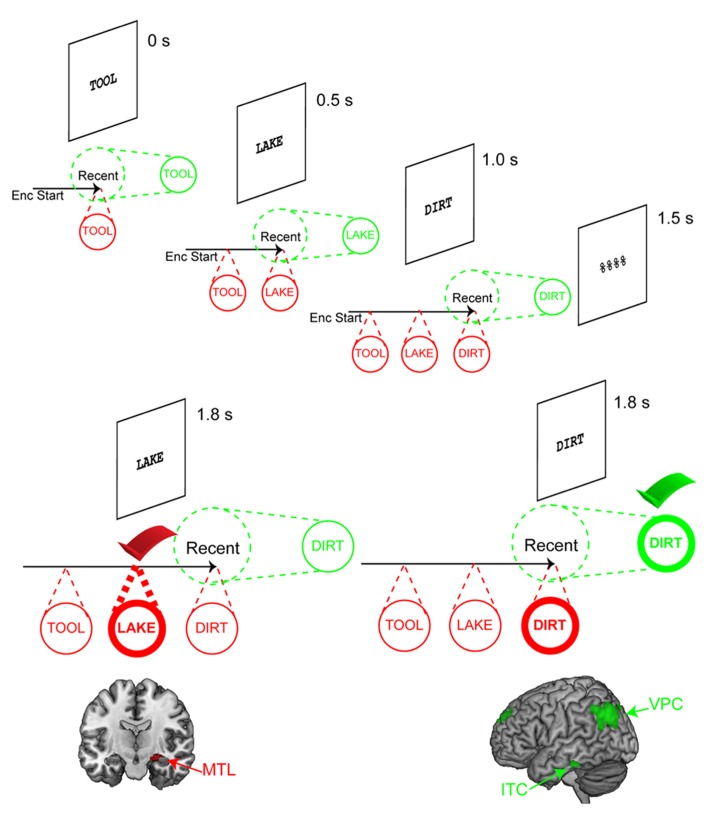
**Assessing representational states via serial positions.** Illustration of the task used by Nee and Jonides (2008) to dissociate the focus of attention from the direct-access region. Three words are sequentially presented followed by a mask and a recognition probe. Timing information depicts the onset of each stimulus relative to the start of the trial. As each memorandum is presented, it is assumed that it becomes the focus of attention. Thus, upon presentation of “TOOL,” “TOOL” is the focus of attention. When “LAKE” is presented, the focus of attention switches to “LAKE.” As each item is presented, it is bound to the temporal context of the trial. At the end of encoding, the last word, “DIRT,” is the focus of attention. Two kinds of probes are depicted. The left depicts a scenario where “LAKE” is the probe. This activates its corresponding representation. The bindings to the trial context, maintained by the direct-access region, verify that “LAKE” is an old probe resulting in a match decision. This elicits activation in the medial temporal lobe (MTL). The right depicts an alternative scenario where “DIRT” is the probe. Once again, “DIRT” activates its corresponding representation. In this case, however, “DIRT” is the focus of attention, so it can be verified immediately without the need to retrieve contextual information. This elicits activation in inferior temporal cortex (ITC) and ventral parietal cortex (VPC). Activation data adapted from Nee and Jonides (2011).

We began by searching for dissociable neural signatures of the focus of attention and the DAR (Nee and Jonides, 2008). To do so, we adapted the rapid serial item-recognition procedure of McElree and Dosher (1989) which had provided strong behavioral evidence for a distinct, single-item focus of attention. On each trial, three items were presented serially followed by a brief mask and a probe. We assumed that upon presentation of the memory set, each item would be represented in the focus of attention until the arrival of the next memorandum, such that at the end of the trial, the focus of attention should linger on the last item (i.e., most recent item). Since encoding and maintenance demands were identical across all conditions, examining how information was accessed provided a window into different states of maintenance^3^. Thus, probes matching the most recent item should reflect access of the focus of attention while probes matching either of the other two items would reflect access of the DAR (**Figure 2**). Given our model, we predicted that accessing items in the DAR would be accomplished through contextual bindings and thus elicit activation in the MTL. Such contextual retrieval was not predicted to be necessary to access the focus of attention. Since the MTL has traditionally been linked to retrieval from long-term memory (LTM), we chose to keep the memory set within bounds of putative 4 ± 1 capacity limits so that MTL activation during retrieval could not be confounded with retrieval from LTM. Furthermore, the rapid presentation of items and the brief retention interval provided further assurance that retrieval was from STM and not LTM (but see Luck, 2008).

Retrieval of information outside of the putative focus of attention elicited activation in the MTL as predicted. Furthermore, the amount of MTL activation closely tracked retrieval demands. Behavioral data indicated a primacy effect such that the first presented item was retrieved more quickly than the second item. MTL activation closely mirrored this pattern, activating more for retrieval of the second than the first item. Around the same time as our publication, Oztekin et al. (2009) published very similar findings using a five-item memory set with both item-recognition and judgment of recency tasks. However, in those data, retrieval of the first item (i.e., most distant item, serial position -5) did not elicit activation in the MTL. Such data suggest that MTL activations drop-off for more distant items, perhaps at the limits of a 4 ± 1 capacity of the DAR.

The second point of interest was regions involved in the access of the focus of attention. Our model predicted that the focus of attention was unique in its association with activity in posterior representational cortices. We conjectured that probes matching the focus of attention would elicit increased activations in regions involved in this representation, similar to match-enhancement effects observed in object-sensitive temporal areas in monkeys (Miller and Desimone, 1994). We found activation in the lateral inferior temporal cortex (ITC) when the focus of attention was accessed relative to other items. These areas were anterior to temporal regions that demonstrate object-sensitive activity such as the visual word form area (Cohen and Dehaene, 2004), which we took to mean that the information was represented in a more semantic than visual form consistent with abstraction gradients in temporal cortices (Martin and Chao, 2001). Furthermore, increased correlations were observed between this temporal activation and activation in the ventral posterior parietal cortex (VPC) when the focus of attention was accessed relative to other items. Across other studies, activation in the VPC would prove to be the more reliable marker of access of the focus of attention.

Having established dissociable neural signatures associated with accessing the focus of attention and information outside of the focus of attention, we next sought to establish whether neural patterns of retrieval distinguished access of the DAR and aLTM (Nee and Jonides, 2011). To do so, we used virtually the same paradigm as before, but we increased memory load to six items. We hypothesized that six items would exceed the capacity of the DAR given its putative 4 ± 1 item limit. We reasoned that rapid presentation of items and a brief retention interval would minimize chunking that could otherwise expand the limit to 7 ± 2 (Miller, 1956). Furthermore, we reasoned that at the time of the probe, the DAR would consist of the items most closely linked to the probe context. In this case, the context is temporal so that the most recently presented items up to a capacity limit would be bound via the temporal context to the DAR. Once again, we assumed that the most recent item would be the focus of attention. Behavioral data demonstrated a precipitous drop in retrieval accuracy between the third and fourth most recent items suggesting that approximately three items were linked to the DAR in our paradigm. As a result, we measured activation to probes matching the second and third most recent items as reflective of access to the DAR, with items beyond this limit (i.e., fourth, fifth most recent) reflecting access of putative aLTM.

**FIGURE 3:**
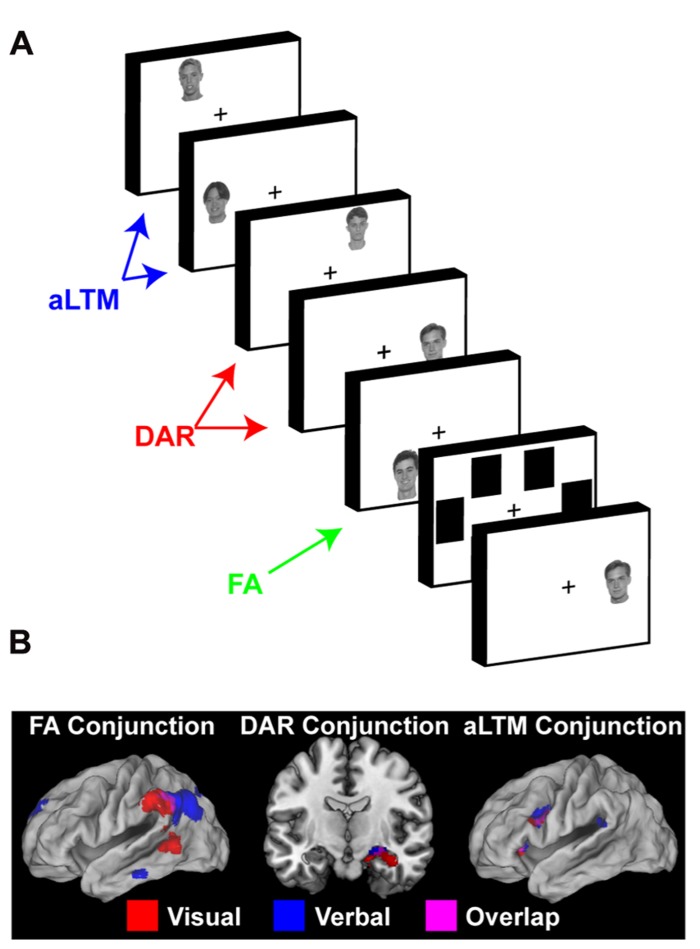
**Neural evidence for a three-state model of memory.** **(A)** The task used by Nee and Jonides (2013) to examine neural correlates of the focus of attention (FA), direct-access region (DAR), and aLTM in visual STM. The task involved sequential presentation of five faces followed by a mask and a recognition probe. In this task, the number of presented items exceeded the capacity of the direct-access region. So, it was assumed that the least recent items (i.e., first presented items) would no longer be contextually bound and be represented in aLTM rather than the direct-access region. Reprinted from Nee and Jonides (2013) with permission from Elsevier. **(B)** Conjunction of results across a six-word version of the paradigm depicted in **Figure 2** (Nee and Jonides, 2011) and the five-face paradigm depicted in **(A)** (Nee and Jonides, 2013). Across both studies, probes matching the focus of attention activated ventral posterior parietal cortex (VPC), probes matching the direct-access region activated the medial temporal lobe (MTL), and probes matching the aLTM activated the ventrolateral prefrontal cortex (VLPFC). In all cases, activations related to a given state were dissociable from those involved in the access of other representational states. This triple dissociation supports the three-state model of memory. Reprinted from Nee and Jonides (2013) with permission from Elsevier.

The results both replicated and extended our prior findings. First, accessing the focus of attention was again associated with activation in lateral ITC, as well as the VPC. In these data, activations in the VPC were substantially more pronounced. Furthermore, compared to accessing the focus of attention, the MTL was more active when accessing the DAR, also replicating our previous results. In addition, the MTL was more active when accessing the DAR compared to aLTM. This latter aspect was surprising given the important role the MTL plays in LTM, but was consistent with patterns suggested by the data of Oztekin et al. (2009). Finally, the ventrolateral prefrontal cortex (VLPFC) showed increased activation for probes that matched the contents of putative aLTM compared to accessing both the focus of attention and the DAR. Taken together, the data demonstrated a triple dissociation: access of the focus of attention invoked VPC activation, access of the DAR involved the MTL, and access of aLTM elicited the VLPFC (**Figure 3B**). In a follow-up study, we repeated the experiment using faces as stimuli (**Figure 3A**; Nee and Jonides, 2013). Here, we tailored our analyses to individually measured capacity estimates, associating retrieval from the DAR to within-capacity items and retrieval from the aLTM to supra-capacity items. The same pattern in VPC, MTL, and VLPFC was observed (**Figure 3B**), suggesting that the three-state model applies in a similar manner across both verbal and visual STM.

While our data support a three-state model of STM, a closely related study produced somewhat discrepant results (Oztekin et al., 2010). This study used a twelve-item serial recognition procedure with words in which the probe consisted of both an old and a new item from which the subject chose the old item. The probe method differed noticeably from our studies in which only one item was presented as the probe and was judged as either old or new (Nee and Jonides, 2008, 2011, 2013). Consistent with our results, accessing the focus of attention (i.e., when the old probe matched the most recently presented item) was associated with activation in the VPC as well as lateral ITC. However, MTL activation was associated with accessing all items outside of the focus of attention (i.e., both DAR and aLTM) and no differences in VLPFC activation were reported. Although activation was numerically greater for probes matching the putative DAR relative to putative aLTM, this difference did not reach significance. Instead, activations in the MTL varied as a function of correct retrieval, with increased MTL activation for appropriately identified old items relative to inappropriately endorsed new items. As a result, these authors settled on a two-state model with a single-item focus of attention contrasting with all other items that vary only quantitatively in memory strength (McElree and Dosher, 1989; McElree, 2006). We speculate that differences between these data and our own are attributable to the two-alternative procedure. First, search demands may have been increased due to the need to select between two probes. In other item-recognition settings, new probes elicit greater activation than old probes in the VLPFC (Nee et al., 2007). Hence, VLPFC activation corresponding to new probes may have muted any existing differences in VLPFC activation between different old probe types. Moreover, the need to distinguish between two items may have increased demands on contextual retrieval. Such contextual retrieval processes are hypothesized to involve the MTL (Eichenbaum et al., 2007). As a result, the two-item probe procedure may have masked differences within the MTL and VLPFC that have been revealed by single-item probe procedures.

Other work has examined the neural correlates of switching the focus of attention. In one study (Lepsien and Nobre, 2007), participants encoded a face and scene into STM. Thereafter, a cue instructed participants to focus attention on either the face or the scene. A subsequent cue asked them to either maintain attention on the currently relevant object, or switch attention to the other object. Inferior temporal areas known to be sensitive to either face or scene processing closely tracked the focus of attention: when attention was on the face, face-sensitive areas were more active. By contrast, when attention was on the scene, scene-sensitive areas were more active. Furthermore, dorsal parietal cortex (DPC) and lateral PFC were active whenever attention was oriented. These data are consistent with the proposal that frontal-parietal areas direct the focus of attention while activity in posterior representational cortices instantiate the items in the focus of attention (Jonides et al., 2008). In a related study (Lewis-Peacock et al., 2012), participants encoded two of three potential categories of stimuli into STM. Machine-learning algorithms were trained to detect the presence of particular categories of information in mind through the pattern of activation across the brain (Norman et al., 2006). Thereafter, participants were cued to one of the encoded categories and responded to a probe. Next, participants were cued with either the same or the other category and responded to a probe. Hence, the cues directed the focus of attention to a particular item/category in STM. Interestingly, only the pattern corresponding to the category in the focus of attention could be detected. Although subjects could successfully switch between items (demonstrating that information about the non-focused item was still present somewhere) the pattern corresponding to the non-focused item could not be detected. These data are consistent with the idea that only the information in the focus of attention is represented by active neural firing.

Recordings from monkeys provide additional insights. In one study, monkeys were sequentially presented with two objects followed by a recognition decision on a matching or non-matching sequence of objects (Warden and Miller, 2007, 2010). Recordings were made in the lateral PFC, with most object-selective cells observed in the VLPFC. After presentation of the first object, object-selective delay activity corresponding to the first object was high, but this object-selective activity was substantially reduced following the presentation of the second object. Instead, object-selective delay activity was high for the second object following its presentation. Hence, the most recently presented object was most substantially represented in PFC neural activity. This is consistent with the idea that the focus of attention lingers on the most recently presented item in serial item-recognition tasks. Interestingly, if monkeys were instructed to recall the sequence, rather than recognize it, a different pattern emerged in the delay interval following the second object. While early delay period activity once again reflected the second object, this activity shifted to reflect the first object later on in the delay (Warden and Miller, 2010). Hence, it appears as though the object represented by the PFC shifted to prepare for recall demands. A further analysis of these recall data revealed a periodicity in PFC activity (Siegel et al., 2009). Population activity in the PFC was found to be synchronized at 3 and 32 Hz. Interestingly, information about each object in STM was maximal at distinct phases of the 32 Hz cycle and this information was modulated by the slower 3 Hz oscillations. The nesting of high frequency oscillations within low frequency oscillations has been hypothesized to be a mechanism of high-speed scanning in STM (Lisman and Idiart, 1995; Jensen and Lisman, 1998). Moreover, 32 Hz corresponds roughly to the estimated rate of human STM scanning (Sternberg, 1966) suggesting a common data rate among primates. Thus, it is tempting to conclude that these rhythms reflect the cycling of the focus of attention among different items in STM to keep them active in preparation for recall.

To summarize, neural data have provided evidence for a three-state model of memory. In serial item-recognition paradigms, a triple dissociation in neural activation has been observed during the access of distinct states of memory (Nee and Jonides, 2011, 2013). Activation in inferior temporal areas and VPC accompany the access of the focus of attention. The focus of attention can be cycled among different representations through the action of frontal regions with the information represented by the focus of attention reflected in different areas of cortex such as ITC (Lepsien and Nobre, 2007). Information is contextually bound by the MTL, providing a means to access items outside of the focus of attention, but within the presently relevant context. This binding in STM is presumed to lay the groundwork for new long-term traces (Schon et al., 2004; Ranganath et al., 2005). Finally, information that has weak or no associations to the currently relevant context can be resuscitated through the action of VLPFC. The VLPFC may work in concert with the MTL to retrieve such content and/or to update appropriate item-context bindings (Nee et al., 2007; Nee and Jonides, 2008). With these data in mind, we now consider each of these potential mechanisms in more detail. Afterward, we consider an updated neural model of memory in light of new findings.

## RELATIONSHIP BETWEEN INTERNAL AND EXTERNAL ATTENTION

Increasingly, the mechanisms governing external selective attention are thought to correspond to mechanisms involved in maintaining information internally (Kane et al., 2001; Chun and Johnson, 2011; Gazzaley and Nobre, 2012). This is in part due to the predictive power of the capacity of STM on performance in tasks requiring selective attention (Kane et al., 2001), the modulatory effect of STM load on selective attention performance (de Fockert et al., 2001; Kim et al., 2005), as well as the high degree of neural overlap in tasks comparing internal and external selection of information (Kuo et al., 2009; Nee and Jonides, 2009). However, much of this research has treated STM holistically. What are the implications of different states of memory on the relationship between internal and external attention?

External attention is often guided by an explicit goal, such as searching for a friend in a crowd. In such situations, it is hypothesized that an attentional template biases search toward goal-relevant information (Desimone and Duncan, 1995). The attentional template is presumed to be maintained in STM. So, this account predicts that the contents of STM bias external attention. There are now numerous examples demonstrating that holding information in STM causes external attention to be captured by visual objects matching the stored contents (see Olivers et al., 2011; Kiyonaga and Egner, 2013; Luck and Vogel, 2013 for recent reviews). For example, when subjects maintain an item in STM for a subsequent recognition test, attention is captured by irrelevant displays containing that item (Downing, 2000). However, attention is not always drawn to memory items. In one study, participants were presented with two items that were targets for visual search (Houtkamp and Roelfsema, 2006). Each target was presented on a different half of the screen. After encoding the items, a search set was presented on one half of the screen directing a search for the target that had been presented on that side (e.g., search for the left target). After reporting the presence or absence of the target, subjects then searched for the other item (e.g., search for the right target). Interestingly, search times were not influenced by the appearance of the irrelevant target as a distractor item (e.g., the right target as a distractor if searching for the left target). Thus, even though subjects had to remember both targets, only the target relevant for the current search influenced attention. Similarly, if subjects are given two items to remember, one as a search target and another as a subsequent item-recognition target, search times are unaffected if the recognition item appears as a distractor (Downing and Dodds, 2004). These examples illustrate that not all items in memory obligatorily become attentional templates. Instead, subjects can constrain search to a single relevant template. This can be accomplished if the attentional template corresponds to the focus of attention (Olivers et al., 2011). By contrast, items relevant only for subsequent operations may be passively maintained in aLTM to minimize their interference with current task goals. Just as items in aLTM do not impact the rate of search of internal information (Oberauer, 2002, 2005), items in aLTM may not impact the rate of search of external information.

Other aspects of the focus of attention mimic patterns observed in external attention. A recent study demonstrated that when the focus of attention was directed to a recently presented item (called “refreshing”), responses to probes of that item presented 100 ms later were slowed (Johnson et al., 2013b). This pattern appears conceptually similar to inhibition-of-return in external attention in which attention is slow to return to a location that was just processed (Posner et al., 1985). Such mechanisms are thought to facilitate search, enabling disengagement of attention from an already processed location/item in order to processes new locations/items. Hence, searches of STM may operate under the same principles.

If the focus of attention corresponds to the attentional template in search, we would expect similar neural activations for internal searches of STM and external searches of the environment. As we reviewed above, switching the focus of attention among items in STM involves top-down control processes in frontal and parietal areas that bias processing in object-sensitive temporal cortices (Lepsien and Nobre, 2007). Very similar patterns are observed when external attention is switched between visually presented faces and scenes (Serences et al., 2004). A recent study directly compared the neural correlates of shifting the focus of attention in STM with shifting external attention (Tamber-Rosenau et al., 2011). This study found highly overlapping activations across both forms of shifting in the DPC and superior frontal sulcus (SFS) – the standard dorsal attention network (Kastner and Ungerleider, 2000; Corbetta and Shulman, 2002). However, a machine-learning algorithm could distinguish subtle differences in the patterns of activation across the DPC and SFS to appropriately classify internal versus external shifts. These data suggest that overlapping but distinct populations of neurons are involved in shifts of internal versus external attention. On the one hand, this result is comforting: if internal and external attention could not be distinguished, we might mistake our memories for percepts (Chun et al., 2011). On the other hand, that both forms of attention exist in the same neural regions enables a high degree of interaction between neurons responsible for internal and external attention. Such an arrangement may maximize the efficiency of the interaction between the focus of attention and external attention, facilitating the ability to search for attention templates in the environment.

Finally, similar neural recruitment underlies target detection of both the focus of attention and attentional template. While the dorsal attention network guides the search process, the ventral attention network reflects the process of detecting the sought-after target (Corbetta and Shulman, 2002). Common recruitment of the VPC in attention and memory has been hypothesized to reflect a common mechanism of attentional capture across both domains (Cabeza et al., 2008, 2012). Consistent with these ideas, we and others have repeatedly demonstrated the involvement of VPC when recognition probes match the focus of attention (Nee and Jonides, 2008; Oztekin et al., 2010; Nee and Jonides, 2011, 2013). Such activations may reflect the capture of attention by items that match the focus of attention in just the same way that attention is captured by targets matching the attentional template.

We have suggested that the focus of attention and the attentional template that guides external search are one and the same. We have further hypothesized that targets for future operations can be relegated to aLTM so as to prevent interference from current goals. At the present, it is unclear how information in the DAR that is not the focus of attention impacts external attention. A recent study demonstrated that subjects can simultaneously search through two colors at once to locate a target (Beck et al., 2012), although search rates are slowed in this case relative to searching through a single color. These data suggest that more than a single item can impact search. It could be the case that simple features such as color can be chunked into a single representation that is then held in the focus of attention as the attentional template. Or it could be the case that the focus of attention cycles between the two items with both bound to the DAR. It is notable that mean search times were about 200 ms longer for dual-cue searches relative to what would be predicted by doubling the search time for single-cue searches. If the focus of attention can be cycled at a rate of 32 Hz as suggested earlier (Sternberg, 1966; Siegel et al., 2009), and the focus of attention cycles between search candidates when searching for two items, then cycling of the focus of attention may account for the additional observed search durations. The estimated number of objects searched was seven per trial, which when multiplied by a constant increase in search rate predicted by 32 Hz cycling would roughly correspond to the observed 200 ms difference. While this consistency is intriguing, future research will be needed to sort this out.

## CAPACITY AND THE DIRECT-ACCESS REGION

The DAR is presumed to bind a limited number of items to a context, thereby enabling context-driven retrieval. It may be natural to assume that neural correlates of the DAR can be revealed by parametrically manipulating maintenance demands. By this logic, the need to maintain more items in STM places greater demands on the DAR to link those items to a context, resulting in more neural activity in brain areas responsible for these processes. Many fMRI studies have used precisely this parametric logic to examine the neural correlates of STM (see Wager and Smith, 2003; Rottschy et al., 2012 for summaries). Such studies converge on a frontal-parietal network with dorsal-ventral differences in frontal activations as a function of material (Rottschy et al., 2012). As a result, these areas may be candidates for the operations of the DAR.

An important limitation of the standard parametric approach is that as STM load increases, so too does difficulty in general. While it is possible that difficulty can be operationalized as STM demand, it is notable that the commonly activated frontal-parietal network is recruited across a variety of other demands, as well. For example, a recent meta-analysis found little to distinguish STM from other cognitive demands in the frontal-parietal network (Niendam et al., 2012). Many of these functions, such as vigilance and inhibition, appear to place minimal demands on contextual binding. So, it is likely that many of these frontal-parietal areas perform rather general cognitive functions, but may not perform the binding operations that underlie the DAR. Noting the commonality between areas involved in STM maintenance and cognitive control, we have hypothesized that frontal regions perform a general selection function with different networks involved according to the domain (e.g., verbal, spatial) of the content selected (Nee et al., 2013). Under this idea, maintaining information in STM involves repeatedly selecting that information (e.g., cycling the focus of attention among items). Although repeatedly selecting content provides a means to maintain information, it does not necessarily contextually bind that information. So, binding may be related to other brain areas.

Previously we suggested that the MTL is involved in contextual binding in STM (Jonides et al., 2008) making it a likely candidate for the operations of the DAR. At first blush, this notion may seem inconsistent with the literature reviewed above. In particular, if the DAR is central to STM and load is one means to drive activity in STM-related networks, why is the MTL not consistently reported in STM tasks? One answer may be that typical STM tasks place low demands on contextual processing. For example, greater MTL activation is observed when object-location bindings are maintained in STM relative to only objects or only locations (Mitchell et al., 2000; Piekema et al., 2006). Hence, the MTL appears to be engaged to a greater degree when contextual information is necessary for successful performance. However, not all associations are MTL-mediated. Piekema et al. (2006) observed that in contrast to its involvement in object-location bindings, object-color associations did not recruit the MTL. These authors speculated that object and color information were already integrated in higher visual areas obviating the need for MTL-mediated binding. By contrast, when multiple features are represented in distant cortical areas (i.e., object-temporal, location-parietal), the MTL may be necessary for binding. Variability in binding demands across paradigms may therefore explain the inconsistent involvement of the MTL in STM.

Another possibility for the inconsistent reports of MTL activation in STM may lie in the competitive dynamics of the DAR. Oberauer (2009) hypothesized that interference limits the capacity of the DAR. By this account, there is competition for item-context bindings such that linking a new item to a context disrupts the bindings that other items have to that context (Oberauer et al., 2012). One possible consequence of such competitive dynamics is that when too many items are maintained in STM, the overabundance of competition severely weakens contextual bindings. That is, when STM is loaded beyond a certain capacity, item-context bindings are dissolved. Such erosion may be reflected neurally in decreased activity in the MTL. In this case, activation in the MTL may show an inverted U-shaped pattern as a function of load: at low loads, MTL activity will be low commensurate with few item-context bindings. As load approaches capacity, MTL activity will rise to a peak at which competition among item-context bindings is manageable. However, at supra-capacity loads, competition drives down the collection of item-context bindings, and MTL activity is reduced. If this account is correct, studies that have investigated STM with the expectation of linearly increasing activity as a function of load may have missed relevant MTL activations.

In fact, there is some evidence for the predicted inverted U-shaped pattern in the MTL. In one study, participants performed an item-recognition task on letters with loads of one, three, or six items (Zarahn et al., 2005). Accuracy was near ceiling at all loads, so load was taken as a proxy for the number of items maintained. Consistent with numerous studies, activation in a frontal-parietal network increased linearly with load. By contrast, the bilateral hippocampi showed a parabolic pattern: activation was lowest at load 1, highest at load 3, but intermediate at load 6. The authors took this as evidence that the MTL is not involved in STM maintenance – at least for letter stimuli. However, if we assume that the capacity of the DAR is 4 ± 1 items (Cowan, 2001), competition for item-context bindings may have driven down MTL activation at load 6 as the number of items exceeded the number that can be successfully bound to a contextsup^4^. A similar pattern was observed in a recent study employing a standard change-detection task (von Allmen et al., 2013). In this study, activations in the intra-parietal sulcus (IPS) rose with the number of items maintained in visual STM, but plateaued when capacity-limits were reached – a pattern that has been observed previously (Todd and Marois, 2004; Xu and Chun, 2006). Activations in the hippocampus also rose with the number of items maintained, showing maximal activation at loads that matched visual STM capacity estimates. However, for supra-capacity loads, activation in the hippocampus decreased, again showing an inverted U-shaped pattern across load. Taken together, these data indicate that the MTL does, in fact, track the number of items maintained in STM up to a capacity-limit. When this limit is reached, MTL activation drops, potentially due to the interference of item-context bindings through competition.

Our hypothesis about the central role of the MTL in STM contradicts the classic view that the MTL is critical for LTM, but not STM. This view is largely supported by data demonstrating impaired LTM, but intact STM, in patients with MTL damage (Scoville and Milner, 1957; Cave and Squire, 1992). However, a number of more recent studies have demonstrated that patients with MTL damage show worse performance than matched controls when tested with STM tasks requiring the maintenance of item-context bindings (Hannula et al., 2006; Olson et al., 2006; Finke et al., 2008; Pertzov et al., 2013). In an elegant study, healthy controls and patients with a treatable form of autoimmune encephalitis that targets the hippocampus performed two experiments requiring the recall of item-context bindings (Pertzov et al., 2013). In the first experiment, one or three objects were simultaneously presented at different locations followed by a brief delay and then two probe objects – one old and one new. Participants were required to identify the old object (identification task). Thereafter, participants were required to drag the identified object to its originally presented location (localization task) using memory of item-location bindings. Patients showed identical performance to controls in the identification task at both loads and the localization task with only one item. However, patients were impaired in the localization task with three items. Further analysis into the nature of the impairment revealed that patients erroneously dragged objects into the locations in which other objects had appeared on the trial. That is, patients “swapped” the object-location bindings and did so twice as often as matched controls. In a second experiment, participants were presented with colored bars in different orientations. Thereafter, a probe bar was presented which was to be rotated to match its originally presented orientation. The same pattern held: patients erroneously rotated the probe bar to a different object’s orientation more often than controls. Finally, one of the patients was re-tested several times after intravenous immunoglobulin injections were administered to treat the encephalitis. Little improvement was observed in the second testing 5 months post-treatment indicating a modest re-test benefit at best. However, performance improved dramatically and approached the performance of controls 10 and 25 months post-treatment. Hence, treatment appeared to restore the item-context binding functions of the MTL. Together, these data demonstrate strong evidence that the MTL is essential for maintaining item-context bindings even in STM.

The above data indicate dual mechanisms of maintenance in STM. On the one hand, a frontal-posterior network selects items and cycled-selection underlies item-based maintenance. On the other hand, the MTL maintains item-context bindings in STM. We suggest that although the former has traditionally dominated discussion of STM, it is the latter that forms the basis of the DAR. Moreover, these mechanisms are likely to be complementary and interactive. For example, if an item representation is lost through failed frontal-posterior maintenance, it can potentially be recovered through contextual bindings. How might such interactions occur?

In a meta-analysis of tasks involving cognitive control over STM, we observed that different frontal networks are engaged depending upon the type of controlled content (Nee et al., 2013). In particular, dorsal frontal areas were consistently activated by control over spatial STM, whereas the VLPFC was engaged in control over object and verbal STM. We hypothesized that this dorsal-ventral dichotomy reflected an extension of the well-characterized dorsal/spatial, ventral/object distinction observed in posterior cortices (Ungerleider and Mishkin, 1982) into the frontal lobes (Levy and Goldman-Rakic, 2000). Similar conclusions have been reached on the basis of lesion (D’Esposito and Postle, 1999; Muller and Knight, 2006) and transcranial magnetic stimulation data (Mottaghy et al., 2002). The essential idea is that frontal areas are involved in top-down selection (Miller and Cohen, 2001) where the form of the selection is dictated by the areas with which the frontal lobes communicate. Dorsal frontal areas select spatial content because they are connected to parietal areas that represent space. Ventral frontal areas select identity content because they are connected to temporal areas that represent objects. Frontal areas are also connected to the MTL (Goldman-Rakic et al., 1984). Through these connections, the PFC may interact with the contextual bindings that underlie the DAR.

One manipulation that may modulate PFC-MTL communication is STM load. As more items are maintained in STM, the likelihood that an item will be lost increases. Thus, there may be greater demand to reinstate lost items through MTL-mediated contextual retrieval. Consistent with these ideas, there is some evidence that functional connectivity between the PFC and MTL increases with STM load (Rissman et al., 2008; Finn et al., 2010). One study examined connectivity among the PFC, MTL, and fusiform face area (FFA) while participants maintained one to four faces in STM (Rissman et al., 2008). Here, activation in the FFA was assumed to be a proxy for the representation of faces in STM. The study revealed three inter-related findings. First, as load increased, the correlation between the FFA and MTL increased. This is consistent with increased communication between the MTL and FFA to form contextual bindings. Second, as load increased, the correlation between the PFC and FFA decreased. Third, as load increased, the correlation between the PFC and MTL increased. Putting these latter two findings together suggests that with increased loads, the PFC shifts from selecting the items directly (i.e., PFC to FFA) to selecting the items through their context (i.e., PFC to MTL). This could occur if item-based information is lost with increased loads due to decay or interference thereby necessitating contextual-retrieval to reinstate the lost information. Hence, these data illustrate a dynamic interplay between the PFC and the DAR. It appears that the PFC can flexibly select different forms of content, be they items or contextual bindings, with different demands dictating the form of content selected.

## MEDIAL TEMPORAL MECHANISMS OF MAINTENANCE

We reviewed evidence that the PFC can cycle through items in STM (Siegel et al., 2009), potentially forming a basis for the maintenance of information. Similar dynamics have been revealed in the MTL (Axmacher et al., 2010) indicating that the PFC is not alone in its capacity for maintaining items. In particular, it has been hypothesized that individual gamma cycles nested within slower theta rhythms reflect the cycling of individual items within a context (Lisman and Jensen, 2013). Evidence for this hypothesis draws from both LTM and STM, and data recorded from rodents, monkeys, and humans. Here, we focus on the human evidence in STM.

Important insights into neural mechanisms of STM maintenance in humans have been revealed by intracranial EEG (iEEG). Two studies have demonstrated that as the number of items held in STM increases, gamma activity in the MTL increases (Axmacher et al., 2007; van Vugt et al., 2010). These patterns have been demonstrated using both faces and letters as stimuli, and they suggest that the number of items held in STM may be reflected in the MTL via gamma power. Moreover, direct comparison of the same STM task using iEEG and fMRI revealed that fMRI activation in the MTL increased as a function of load in similar ways to gamma activity in iEEG (Axmacher et al., 2007). Hence, these data established a correspondence between iEEG and fMRI in the MTL. Finally, a recent study demonstrated increased cross-frequency coupling between gamma and theta frequencies during STM maintenance compared to baseline (Axmacher et al., 2010). In this study, gamma amplitude increased at the peak of the theta phase suggesting that theta activity plays an important modulatory role on gamma activity, consistent with a gamma-theta/item-context association. Interestingly, at all loads, the ratio between gamma and theta frequency remained constant at ~4. This held true even as theta frequencies slowed significantly with increasing loads. While it is possible that this ratio is a coincidence, it bears mentioning that it resembles the presumed 4 ± 1 capacity of STM (Cowan, 2001). This leads to the intriguing possibility that the ratio of gamma to theta frequencies in the MTL determines the capacity of the DAR.

Additional evidence for the relationship between the MTL, theta oscillations, and STM comes from a study that compared STM for relational versus non-relational scenes (Cashdollar et al., 2009). This study used magnetoencephalography (MEG) to study theta activity in patients with hippocampal sclerosis, patients with temporal epilepsy without hippocampal damage (i.e., a control group), and healthy controls. First, patients with hippocampal sclerosis performed more poorly on tests of relational STM compared to both control groups, but they performed normally on tests of non-relational STM. These data are consistent with prior studies demonstrating impaired relational STM in patients with MTL damage (Hannula et al., 2006; Olson et al., 2006; Pertzov et al., 2013). Furthermore, on a surprise delayed recognition test, both control groups recognized relational scenes better than non-relational scenes. However, patients with hippocampal sclerosis did not show this effect. Such effects are consistent with the idea that MTL-mediated STM (e.g., relational STM) lays the groundwork for LTM (Schon et al., 2004; Ranganath et al., 2005), but this relationship is absent if the MTL is damaged. In terms of neural effects, both control groups demonstrated increased occipital-temporal theta coupling during the maintenance of relational STM, but increased frontal-parietal theta coupling during the maintenance of non-relational STM. Patients with hippocampal sclerosis showed the latter effect, but not the former, suggesting that occipital-temporal theta coupling is MTL-mediated. These data are consistent with the idea that frontal-parietal areas are important for item-based STM, but the MTL is critical for relational STM. Furthermore, these data suggest that the MTL binds visual object relations through synchronizing theta activity in posterior cortices.

Taken together, the data provide insights into how the MTL maintains item-context bindings. The MTL coordinates activity in cortical areas through theta oscillations. This synchronous activity in cortical regions provides a means to bind information represented in respective cortical areas (e.g., an object and a location). Individual bindings are then represented by gamma activity that is nested within theta oscillations. The number of bindings that the MTL can maintain is thus limited by the number of gamma cycles that can be nested within theta, a ratio that should reflect the capacity of the DAR. If more items are loaded into the DAR than can be nested within unique phases of theta, it could be the case that synchronicity is disrupted, thus providing a mechanism for interference.

## A NEURAL THREE-STATE MODEL OF MEMORY

**FIGURE 4:**
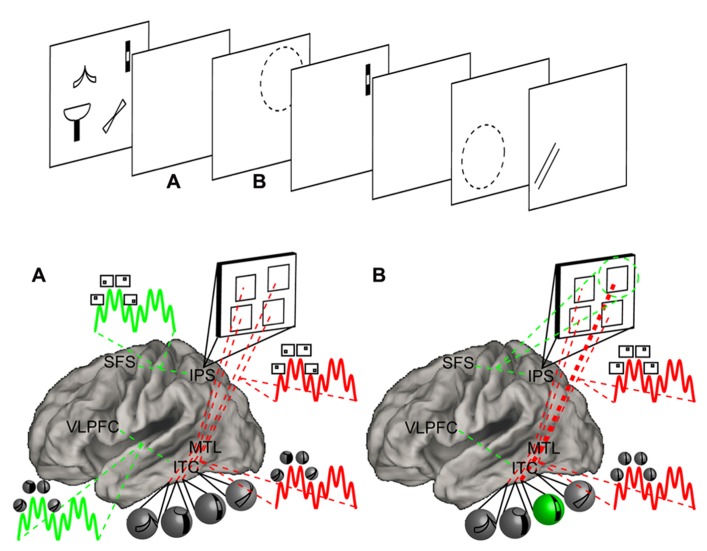
**Neural three-state model of memory.** Top: a hypothetical task requiring the maintenance of visual objects and attention shifting among them. Four objects are presented and encoded into STM. Following a retention interval, a cue directs the focus of attention to one of the objects. A recognition probe is presented in the cued location requiring a match/non-match decision. This is followed by another retention interval, a second cue, and a second probe. Bottom: a model demonstrating relevant areas of the brain and hypothesized psychological and neural processes. The model has been simplified for depictive purposes. For full details, consult the text. Mechanisms associated with the focus of attention are depicted in green while mechanisms associated with the direct-access region are depicted in red. Object information is presumed to be represented in inferior temporal cortex (ITC) while spatial information is presumed to be represented in the IPS. Each of these posterior areas is connected to a corresponding frontal area. The IPS is connected to the SFS, a region that is commonly referred to as the frontal eye fields. The ITC is connected to the ventrolateral prefrontal cortex (VLPFC). Each of these frontal areas selects information represented in respective posterior areas. Finally, the medial temporal lobe (MTL) is connected to both the IPS and ITC and synchronizes their activity. **(A)** During the retention interval, the MTL synchronizes the activity of the IPS and ITC in the theta range. Within each theta cycle, stimulus-specific neurons fire in the gamma range. Thus, gamma activity nested within theta activity reflects the cycling of items within a set. Moreover, item-location bindings are implemented by MTL-mediated synchronized activity in the ITC and IPS. The connections and synchrony correspond to the direct-access region. At the same time, the VLPFC acts upon the ITC to support item-based maintenance. Here also, individual items are nested within oscillatory activity. These mechanisms periodically maintain the activity corresponding to each item in STM and correspond to the focus of attention cycling among the direct-access region. The SFS performs a similar function in concert with the IPS to cycle among locations. **(B)** When a cue directs attention to a spatial location, cycling ceases. Instead, the SFS fixates on the cued location thereby forming the focus of attention. Sustained spatial attention then activates the corresponding object through connections established by previous synchrony. While attention is sustained, only the attended location and corresponding object are instantiated by active neural firing. The synchronous activity between neurons corresponding to location representation and object representation strengthen the bindings between them (thickened red line) potentiating future cued retrieval.

Having reviewed the relevant literature, let us now return to fleshing out neural mechanisms that can account for a three-state model of memory. This model is depicted in **Figure 4**. First, we hypothesize that frontal areas are responsible for top-down selection. Information that is selected depends upon the nature of the region with which frontal cortex communicates. Frontal control over dorsal parietal areas provides a basis for selecting spatial information, frontal control over ventral temporal areas provides a basis for selecting object information, and frontal control over the MTL provides a basis for selecting contextual information. Given that different frontal areas are connected with each of these more posterior areas, it is likely that dissociable frontal regions are involved in selecting different content. The act of selecting information is thus a means for directing the focus of attention. A notable consequence of this formulation is that the focus of attention may be flexibly deployed to an object, a location, or an entire context. If frontal-posterior networks act independently from one another, then each of these forms of content can be selected in parallel thereby conferring a distinct focus of attention for each form of content^5^. By such mechanisms, the model can account for patterns of interference between two attention-demanding tasks performed on the same type of content, but little interference between two attention-demanding tasks performed on different types of content (Logie et al., 1990; Woodman and Luck, 2004). These frontal areas that are involved in content-selection are hypothesized to be coordinated by other more rostral frontal areas forming a hierarchical relationship (Badre and D’Esposito, 2009; Nee et al., 2013). Such coordination is likely to be necessary in dual-tasking scenarios creating a dual-task cost even if each single demand engages a distinct frontal system (Woodman et al., 2001). This idea preserves the notion that the focus of attention is fixated on a single chunk at a given time, but suggests that a separate focus exists for each form of content. Furthermore, multiple “items” may be selected indirectly through selecting a context. This framework may resolve apparent discrepancies regarding how many items can “fit” into the focus of attention (e.g., Gilchrist and Cowan, 2011).

Items are associated to a context through the coordination of multiple cortical sites by the MTL. This provides a mechanism for item-context bindings forming the basis of the DAR. Individual bindings are reflected by activity in the gamma frequency, which are nested within theta oscillations (Lisman and Jensen, 2013). Capacity is predicted to be reflected by the ratio of gamma to theta frequencies – the more gamma cycles that fit within distinct phases of theta, the more bindings can be maintained (Axmacher et al., 2010). Moreover, coordinated activity provides a basis for Hebbian learning (Hebb, 1949). That is, MTL-coordinated neural firing during the maintenance of information in STM leads to new LTM (Schon et al., 2004; Ranganath et al., 2005). When coordinated activity ceases or is disrupted, bindings are no longer considered to be actively maintained. In this case, rapid synaptic potentiation resulting from previous neural synchrony allows item-context bindings to be reinstated.

What distinguishes the DAR and aLTM is that the focus of attention cycles upon the former to maintain bindings. When the focus of attention is fixed upon a particular representation, and is thus not cycling, the difference between the DAR and aLTM is largely quantitative in nature. That is, the DAR simply has stronger synaptic potentiation. When the focus of attention is cycling among the contents of the DAR, oscillatory neural firing distinguishes the DAR from aLTM. When a set of bindings becomes irrelevant, the focus of attention no longer cycles among them and those bindings become aLTM. Due to rapid synaptic potentiation, these bindings are primed and can lead to proactive interference thereby providing behavioral signatures for aLTM. Thus, rapid synaptic mechanisms are responsible for aLTM.

To make the model and its predictions concrete, let us consider an example task (**Figure 4**). Participants are presented with an array of simple objects. Sometime thereafter, a cue indicates that one of the objects will be the target of an upcoming probe. Next, a probe appears at the cued location and participants indicate whether the probe object matches the cued sample object. Thereafter, another cue appears followed by another probe. Thus, on each trial, participants must keep multiple items active (e.g., using the DAR) and switch attention among them (e.g., using the focus of attention) in order to make appropriate decisions.

We will begin by assuming that spatial information is represented in the IPS and object information is represented in ITC. (As an aside, if presentation was sequential rather than simultaneous (Xu and Chun, 2006), the IPS may instead represent order information (Marshuetz et al., 2006)). Our model predicts that there are distinct frontal selectors for each type of information. The SFS selects spatial information from the IPS while the VLPFC selects object information from ITC. Simultaneously, the MTL synchronizes the activity in the IPS and ITC. Frontal areas continually select their respective types of information and may themselves be synchronized through other frontal areas (e.g., dorsolateral PFC or frontopolar cortex). This synchronized cycling between frontal and posterior areas forms the basis of maintenance of item and location information, while the synchronized cycling of the MTL and posterior areas forms the basis of maintenance of item-location bindings. Upon presentation of the cue, attention is directed to the cued location. In this case, the SFS will bias a particular location representation in the IPS. Through its connections to the MTL, the object bound to this location will also be biased. The associated object then becomes the attentional template to which the probe object will be compared. Continuous firing, presumably in the gamma band (Fries et al., 2001; Gregoriou et al., 2009), would then correspond to the location and object in the focus of attention. As a single item is focused, cycling among other items ceases. Thus, items outside of the focus of attention will no longer be associated with active neural firing. However, after responding to the probe, the focus of attention is disengaged, putatively through some inhibitory process (Oberauer et al., 2013; Johnson et al., 2013b), and information that was outside of the focus of attention is retrieved. This is done by exploiting rapid synaptic potentiation of frontal areas to posterior areas on the one hand, and the MTL to posterior areas on the other. For example, if an object was lost due to failed potentiation between the VLPFC and ITC, that item may be retrieved indirectly from SFS to IPS to MTL connections which then re-active the appropriate object through its associated location. Finally, upon presentation of the second cue, the focus of attention again selects a single object through its location. If the second cue was the same as the first, this selection should be facilitated. This is because of the strengthened binding between the object and location that resulted from those representations recently being synchronized in the focus of attention. Notably, recent empirical data support the notion that the focus of attention strengthens item-context bindings (Rerko and Oberauer, 2013). Thus, the focus of attention confers two processing benefits: one due to active firing that makes a representation amenable to further computation, and a second due to strengthened bindings that makes recently focused items easier to retrieve.

It is instructive to consider the impact of MTL damage on the modeled task. Once again, upon presentation of the sample, the IPS will represent spatial information, and the ITC will represent object information. Each information-type will be maintained by frontal areas through cycled selection. However, without the MTL, posterior representations will no longer be directly bound through MTL-mediated synchrony. Nevertheless, some synchrony may occur. This could happen if different frontal areas operate at the same frequencies. Since object and location information were presented simultaneously, this may set distinct frontal-posterior networks to the same initial clock. This implicit synchrony may persist for a short time, but due to stochastic processes, greater and greater degrees of asynchrony would be predicted as time passes. Furthermore, the more items maintained, the greater the demand for precise phase synchrony to minimize interference, and the less likely that this sort of auto-synchrony would be effective. Such an account provides a potential explanation for why STM performance can be spared at short intervals and small loads even when bindings are necessary (Jeneson et al., 2010, 2012). Furthermore, this account suggests that location and object information can be independently spared, while joint bindings are confused when the MTL is damaged (Pertzov et al., 2013).

This model is purposely ambitious to provide a number of avenues of future investigation. One emphasis is on neural interactions. Numerous areas of the brain are hypothesized to be involved in STM, and fMRI data have convincingly localized these areas. Such data have revealed that STM draws upon regions of the brain traditionally associated with attention, as well as LTM, presenting an interlocked picture of these domains. However, to understand STM more thoroughly, dynamic aspects of shifts between representational states will need to be explored. This will involve charting out interactions between frontal areas and targets in posterior cortices and the MTL as information is shifted among different representational states. In this vein, recent work using monkey neurophysiology has demonstrated intriguing shifts in representational states in the PFC (Stokes et al., 2013). How such shifts are mediated by interactions among brain regions remains a mystery. However, the hypotheses raised here may provide an important beacon for directing such research.

A number of mechanisms remain underspecified in the model. Some contextual bindings mediated by the MTL are easy to conceptualize such as the binding of objects and space. However, the distinction between the DAR and aLTM is predicated on knowledge of the bindings relevant for the current temporal context. This suggests that in addition to object-location bindings, the MTL will also need to establish temporal bindings. Some work has suggested that the temporal context is represented in frontal areas so that item-context associations are formed through the interaction of the PFC, MTL, and temporal cortex (Polyn and Kahana, 2008; Sederberg et al., 2008). It is also possible that item–item associations are formed through interactions among simultaneously active representations within a given area of cortex (e.g., multiple objects in ITC). Such associations may provide an additional route to distinguish information that is currently relevant. If such associations can be formed within a localized cortical area, they may not require MTL mediation. Hence, future work that refines the meaning of “context,” the different sort of associations that the brain represents, and how these associations are formed will provide important insights for future models. Moreover, the present model is merely descriptive in nature. The feasibility of these descriptions and mechanistic predictions will be well-served by computational formalism.

## Conflict of Interest Statement

The authors declare that the research was conducted in the absence of any commercial or financial relationships that could be construed as a potential conflict of interest.

## Footnotes

1 The term “working memory” is also commonly associated with the maintenance processes to which we refer. However, the term “working memory” is strongly tied to a particular theory of STM and we use the more theory-neutral term “STM” here.

2 Some authors consider only those representations associated with active maintenance when discussing short-term or working memory (Luck and Vogel, 2013). While such a definition is useful, we also consider passive representations because information is often transitioned from active to passive states and back during ongoing cognition. Since we are interested in the retention system that is used in the service of ongoing cognition, we admit both active and passive representations in our considerations.

3 The mapping of fMRI activation, retrieval, and information representation is potentially complex. In some cases, activation may reflect the retrieval process. In other cases, activation may reflect information representation. In still others, the two phenomena may be confounded such as the case with bindings when the information that is represented (bindings) is also the means to retrieval (items are retrieved through their contextual bindings). For the purpose of experimentation, we were merely interested in identifying dissociations, or lack thereof, to provide evidence for distinct states. The essential logic was that if different neural regions are involved in accessing different putative states, this entails that the states are qualitatively distinct. This, in itself, does not rely on, nor speak to, the functional roles of observed activations. Interpretation of the meaning of the observed activations is based upon our hypotheses and prior knowledge of the roles of various neural regions in cognition.

4 One might wonder why performance was near ceiling at load 6 even despite the decrease in MTL activation. As will be made clear when we discuss our model later, we hypothesize two parallel systems of maintenance: a frontal-posterior system involved in item-based maintenance and a MTL-posterior system involved in maintaining contextual bindings. In an item-recognition task, the demand on contextual processes is minimized and performance is anticipated to be preserved in such a task even if MTL-mediated contextual processes are down-regulated. This is because frontal-posterior networks are sufficient for the maintenance of strictly item-based information.

5 The term “attention” is often associated with the spatial selection mechanism that is, in turn, associated with dorsal networks (i.e., the dorsal attention network). From our perspective, attention corresponds to the act of selection. So, whereas the ventral selection system that we elaborate may not commonly be described as an attentional network, we believe that, functionally, it serves the same purpose. Hence, the focus of attention that we describe corresponds directly to the selection of information of different sorts (spatial, object, or context) whereas the traditional attention literature may speak more specifically to the spatial focus of attention.
